# SRC-2 Coactivator Deficiency Decreases Functional Reserve in Response to Pressure Overload of Mouse Heart

**DOI:** 10.1371/journal.pone.0053395

**Published:** 2012-12-31

**Authors:** Erin L. Reineke, Brian York, Erin Stashi, Xian Chen, Anna Tsimelzon, Jianming Xu, Christopher B. Newgard, George E. Taffet, Heinrich Taegtmeyer, Mark L. Entman, Bert W. O’Malley

**Affiliations:** 1 Department of Molecular and Cellular Biology, Baylor College of Medicine, Houston, Texas, United States of America; 2 Lester and Sue Smith Breast Center, Baylor College of Medicine, Houston, Texas, United States of America; 3 Sarah W. Stedman Nutrition and Metabolism Center, Duke University Medical School, Durham, North Carolina, United States of America; 4 Department of Medicine, Division of Cardiovascular Sciences, Baylor College of Medicine, Houston, Texas, United States of America; 5 Department of Internal Medicine, Division of Cardiology, The University of Texas Medical School at Houston, Houston, Texas, United States of America; University of Geneva, Switzerland

## Abstract

A major component of the cardiac stress response is the simultaneous activation of several gene regulatory networks. Interestingly, the transcriptional regulator steroid receptor coactivator-2, SRC-2 is often decreased during cardiac failure in humans. We postulated that SRC-2 suppression plays a mechanistic role in the stress response and that SRC-2 activity is an important regulator of the adult heart gene expression profile. Genome-wide microarray analysis, confirmed with targeted gene expression analyses revealed that genetic ablation of SRC-2 activates the “fetal gene program” in adult mice as manifested by shifts in expression of a) metabolic and b) sarcomeric genes, as well as associated modulating transcription factors. While these gene expression changes were not accompanied by changes in left ventricular weight or cardiac function, imposition of transverse aortic constriction (TAC) predisposed SRC-2 knockout (KO) mice to stress-induced cardiac dysfunction. In addition, SRC-2 KO mice lacked the normal ventricular hypertrophic response as indicated through heart weight, left ventricular wall thickness, and blunted molecular signaling known to activate hypertrophy. Our results indicate that SRC-2 is involved in maintenance of the steady-state adult heart transcriptional profile, with its ablation inducing transcriptional changes that mimic a stressed heart. These results further suggest that SRC-2 deletion interferes with the timing and integration needed to respond efficiently to stress through disruption of metabolic and sarcomeric gene expression and hypertrophic signaling, the three key stress responsive pathways.

## Introduction

Heart failure results after a culmination of several adaptive changes in the heart initiated from the onset of stress, which include activation of cellular signaling cascades, translational and post-translational regulation of proteins, and altered gene expression profiles. Of these, changes in the gene expression profile of the heart have received extensive attention. As such, many studies have compared gene expression profiles in human failing hearts in an effort to uncover key regulators of the stress response [1], [2], [3], [4], [5], [6], [7]. While cardiac stress often induces similar functional changes in molecular pathways, analysis of gene expression profiles of stressed hearts has revealed very little heart-to-heart overlap in distinct molecular targets altered during cardiac stress. However, Asakura, and Kitakaze recently compiled several human microarray data sets of normal and failing hearts to gain insight into commonly disrupted genes in the cardiac stress response [1]. Their work highlights a transcriptional regulator previously uninvestigated in the heart, SRC-2, as one of only 107 genes found to be altered in more than one data set, suggesting that SRC-2 could be a significant player in the cardiac response to stress [1].

The cardiac stress response requires integrated signaling of several regulatory pathways in order to maintain function under the stress conditions such as increased workload. Three of the main pathways important for this response are the metabolic, sarcomeric, and hypertrophic pathways. For the metabolic and sarcomeric pathways, many of these changes are driven through extensive changes in gene expression that result in a profile that resembles genes expressed strongly during fetal development and is thus described as the “fetal gene profile” [8], [9].

Metabolic changes are driven by an increased demand for fuel, which results in increased glucose use and a corresponding increase in glycolytic gene expression [8]. This increased glucose use is initially in addition to the prevailing use of fatty acid in the adult heart, but under prolonged stress will become the primary source of ATP [9]. Less is understood about the prevailing cause of sarcomeric remodeling; however, extensive changes in both myosin and actin isoforms is routinely observed and thought to play a role in maintaining contractile force [8], [10].

These gene expression changes are ultimately controlled through changes in transcription factor activity and/or expression. Numerous studies have identified several transcription factors that play key roles in regulating the metabolic, sarcomeric, and stress response programs in the heart [9]. One major candidate group is the PPAR family of nuclear receptors and their associated coregulators PGC-1 α and β, which are well-characterized regulators of the cardiac fatty acid metabolic program [11], [12], [13], [14]. Another class of transcription factors have been shown to form several interchanging complexes and work in concert with each other in response to various stresses to drive gene expression [9] and include factors such as SRF, GATA4, MEF2, Hand2, Nkx2.5, and Tbx5. Work from numerous laboratories has highlighted key roles for these factors during stress and for regulation of key stress responsive gene changes ([15], [16], [17], [18], [19]).

The hypertrophic signaling response is stimulated by hoop stress placed on the ventricle walls and results in increased ventricle wall thickness, driven largely through increased cellular size via upregulation of protein translation. In response to TAC, protein synthesis is enhanced through activation of mTOR and its downstream targets. A main activator of mTOR is the PI3K/Akt signaling cascade, which is often activated in concert with increased glucose uptake into the cell during the initial stress onset [20], [21]. Conversely, mTOR activity is inhibited through activation of the metabolic sensor AMPK when ATP levels are depleted [22], [23].

Additionally, there is also a transcriptional component to the hypertrophic response, driven through elegant regulation of several signaling cascades and transcription factor expression and activity. For example, activation of GATA-4 activity through phosphorylation at S105 has been shown to be required for stress-induced hypertrophy in adult mice. This site is phosphorylated by Erk1/2, which are activated during cardiac stress [24]. Another regulatory pathway important for transcriptional regulation of hypertrophy is that of calcineurin signaling, which involves modulation of another key cardiac transcription factor NFAT. Genetic models of calcineurin inhibition also show resistance to stress-induced hypertrophy [25].

The steroid receptor coactivator (SRC) family of proteins is well characterized for their ability to coactivate a number of transcription factors in response to cell signals, especially those of the nuclear receptor family. The SRC family members, SRC-1, -2, and -3, share a similar domain structure including an N-terminal basic helix-loop-helix-Per/ARNT/Sim homology domain involved with protein-protein interactions, a central region containing three LXXLL motifs characterized for interaction with nuclear receptors, and two C-terminal activation domains which interact with histone acetyltransferases and methyltransferases to mediate the transcriptional activation activity of the SRCs. Despite a similar structure and biochemical activity, analyses of mouse models with genetic ablation of the SRCs have highlighted several non-redundant biological roles in organ function, development and disease (reviewed in [26]). Interestingly, several studies using SRC-2 KO mice indicate that SRC-2 activity is important for maintaining metabolic homeostasis in several tissues in response to stresses including pregnancy [27], energy deprivation and energy overload [28], [29], [30]. The SRC-2 coactivator has been demonstrated to coordinately regulate pathways in multiple tissues for energy production and function.

Based on these previously published roles for SRC-2 in controlling enzymes of fatty acid and glucose metabolism in other metabolic tissues [28], [29], [30], [31], its recurrent down regulation in human heart failure [1], and the large transcriptional component involved in the cardiac stress response, we investigated a role for SRC-2 in controlling gene expression in the heart. These investigations including genome-wide analysis, targeted expression analyses, and functional studies before and during pressure-overload induced cardiac stress reveal a novel role for SRC-2 in the regulation of cardiac transcription.

## Methods

### Animals

All animal experiments were performed in accordance with and have been approved by the Institutional Animal Care Research Committee at Baylor College of Medicine (Protocol AN-544 and AN-124). The generation of the SRC-2 KO mice has been described previously [27]. Male, age-matched littermates (10–16 weeks old) mice were used.

### Tissue Lysates and Immunoblotting

Frozen hearts isolated from WT and SRC-2 KO animals were homogenized in 1× HLB (25 mM Tris-HCl, pH7.4, 250 mM NaCl, 5 mM EDTA, 1% Triton x-100, 0.1% SDS, 0.1% sodium deoxycholate, 1 mM DTT, 1X protease inhibitor cocktail (Sigma), 1X phosphatase inhibitor cocktail (Roche)) and cleared via centrifugation and the use of a cellulose-acetate column. SDS-PAGE analysis and transfer were performed by standard protocols. Subsequent immunoblotting was performed with the indicated antibodies by standard methods. Secondary antibodies were HRP-conjugated and signal detection was performed using ECL reagent and autoradiography. Quantitation was performed using Image J software (NIH). All quantitation includes correction for changes in background.

### RNA Isolation and qPCR Analysis

For RNA isolation, heart tissue was homogenized and RNA was isolated using the RNEasy kit according to the manufacturer’s protocol (Qiagen). cDNA synthesis was performed using Superscript III according to the manufacturer’s instructions (Invitrogen). qPCR analysis was carried out using the Taqman system with sequence-specific primers and the Universal Probe Library (Roche). Primer sequences are available upon request.

### Transverse Aortic Banding

Hemodynamic pressure overloading was performed in male WT and SRC-2 KO littermate mice ages 10–12 weeks. Transverse aortic banding was performed using previously described methods [32]. Two-dimensional echocardiography images in M-mode were acquired using a Vevo 770 RMV-707B 30-MHz probe (VisualSonics, Toronto, Canada). Cardiac Doppler measurements were acquired with a Doppler signal processing workstation (DSPW, Indus Instruments, Houston TX) using a 10-MHz, 1 mm diameter pulsed Doppler probe. Echocardiography and Doppler measurements were made as previously described [32], [33], [34].

### Lactate Analysis

Heart tissue was homogenized in an acetonitrile-formic acid solution on ice and used for lactate detection via mass spectrometry methods according to previously published protocols [35], [36], [37].

### Microarray analysis

For microarray analysis, 250 ng of RNA isolated from total heart (RNeasy kit, Qiagen) for each sample was labeled using the new standard Affymetrix linear amplification protocol using the 3′ IVT Express Kit. This was reverse-transcribed and cRNA was produced and biotinylated via *in vitro* transcription. A hybridization cocktail containing Affymetrix spike-in controls and 15 µg fragmented, labeled cRNA was loaded onto a GeneChip® Mouse 430 2.0 array. The array was hybridized for 16 hours at 45°C with rotation at 60 rpm then washed and stained with a strepavidin, R-phycoerythrin conjugate stain using the FS 450_0001 Fluidics protocol setting. Signal amplification was done using biotinylated antistreptavidin. The stained array was scanned on the Affymetrix GeneChip® Scanner 3000. The images were analyzed and quality control metrics recorded using Affymetrix Command Console v3. All raw data is available at GEO (http://www.ncbi.nlm.nih.gov/geo/).

### Statistical Analyses

All results are depicted as the mean ± standard error of the mean (SEM). The statistical comparison of different groups was carried out using two-tailed unpaired Student’s *t* test, and differences at or below P<0.05 were considered statistically significant. For mice studies, the experimental number used is indicated in each *Figure Legend*.

For the microarray, experiments were run using Affymetrix MG 430 2.0 chip with 45,101 probesets representing 20,757 unique genes. There were 8 experiments in 2 groups: WT-unstressed –4 experiments and KO-unstressed –4 experiments. QC parameters for all experiments were within the acceptable limits. We used the following software packages for data QC, statistical analysis and presentation of the results: Affymetrix Expression Console (www.affymetrix.com), Partek (www.partek.com), BRB Array Tools (linus.nci.nih.gov/BRB-ArrayTools.html), and dChip (biosun1.harvard.edu/complab/dchip).

Expressions were estimated using the RMA (Multi-Array Analysis) method [38] with Partek software. Differentially expressed genes were found using the RVM (Random Variance Model) t-test, which is designed for small sample size experiments [39]. We used BRB Array Tools software, developed by Dr. Richard Simon and the BRB-ArrayTools Development Team. All genes were included in the comparison. For the genes represented by more than one probeset, we used the most highly expressed probeset. The cutoffs for differentially expressed genes were False Discovery Rate (FDR) = 0.05 [40]. Clustering pictures with heat maps were made with dChip software [41]. Pathway analysis was performed using Gene Set Enrichment Analysis (Broad Institute, www.broadinstitute.org/gsea/index.jsp) on differentially expressed genes with an FDR <0.15.

## Results

### Loss of SRC-2 Results in Extensive Gene Expression Changes in the Heart

**Figure 1:**
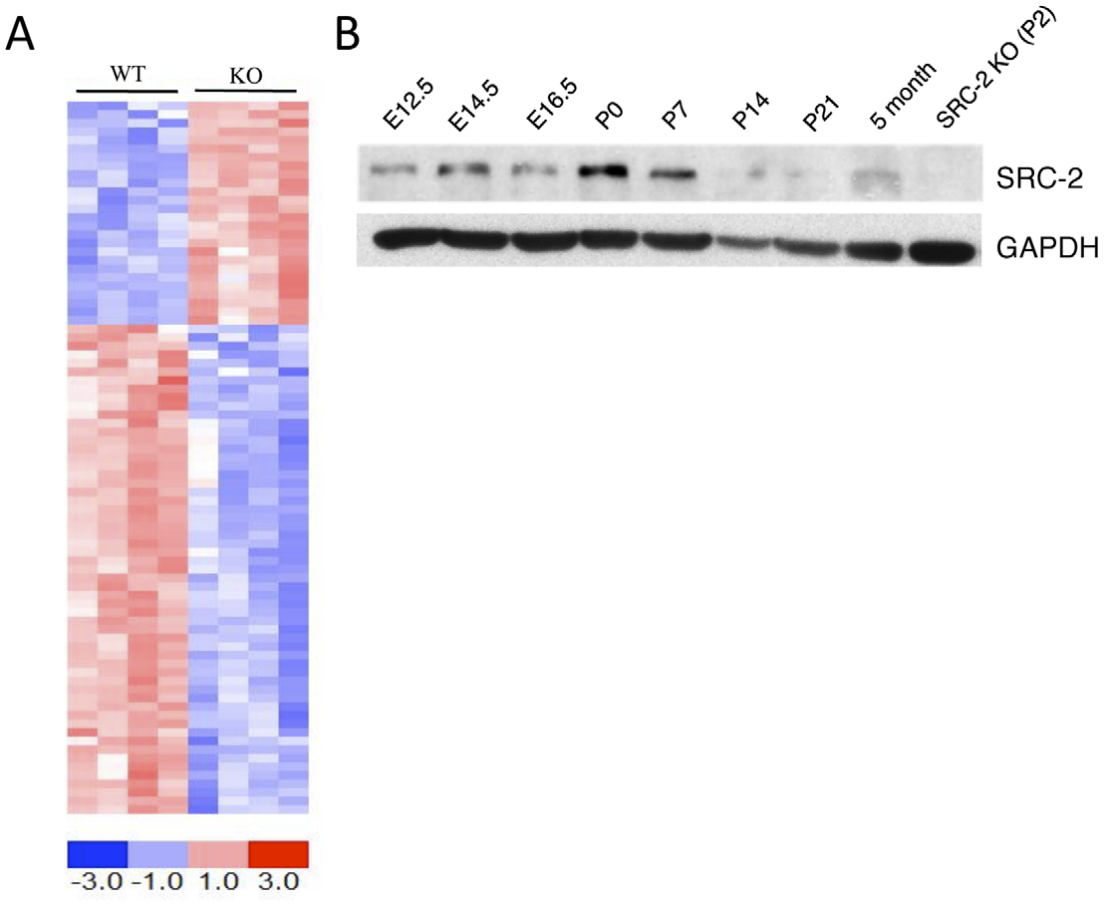
Loss of SRC-2 results in extensive gene expression changes in the heart. *A*, Heatmap and list of genes significantly up and downregulated in WT and SRC-2 KO hearts at FDR<0.05. Each column represents RNA isolated from an individual mouse (WT n = 4, KO n = 4). Rows correspond to gene IDs as listed in Supplemental Table 1. *B*, Immunoblot for SRC-2 in heart tissue lysates made from mouse hearts harvested at the indicated ages of development. GAPDH is used as a loading control.

Recent data suggest that dysregulation of SRC-2 expression may be a common event during cardiac failure [1]; however, little is known about the role of SRC-2 in regulating cardiac gene expression. As such, we used genome-wide profiling to gain insight into the activity of SRC-2 through a comparison of gene expression profiles of WT and SRC-2 KO heart tissue (Figure 1A and Table S1). Analysis revealed distinct gene expression sets that are either up or down regulated in hearts lacking SRC-2. Pathway analysis of altered genes revealed changes in genes involved in many aspects of cardiac function including cell-cell interactions, metabolism, and cell structure, several of which are subject to extensive transcriptional modification during the cardiac stress response (Table S2).

### Ablation of SRC-2 Results in a Cardiac Metabolic Profile Favoring Glycolysis

**Figure 2:**
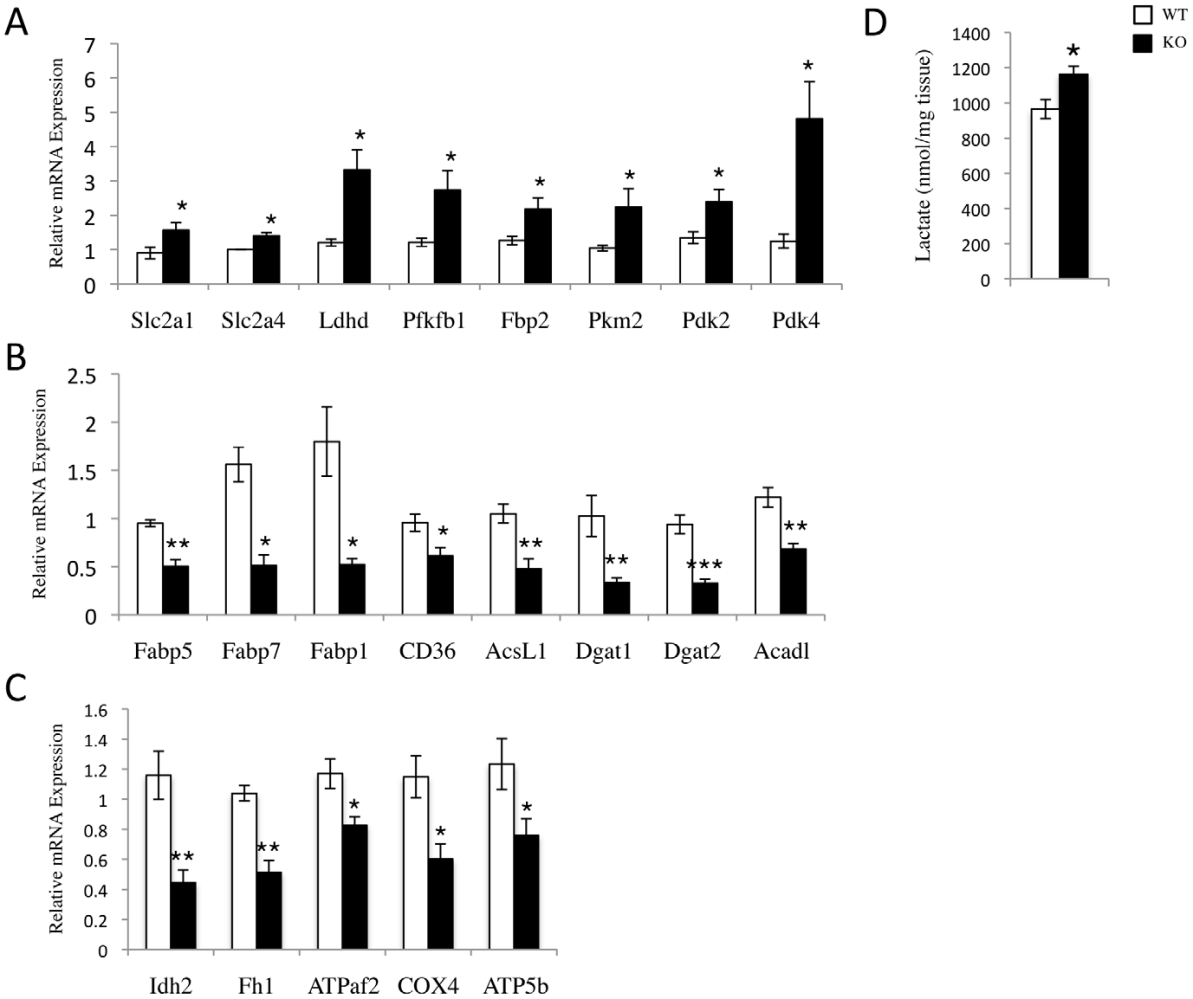
Ablation of SRC-2 in the mouse mimics the metabolic gene expression of a stressed heart. *A*–*C*, Quantitative PCR analysis (qPCR) of gene expression of the indicated gene involved in glycolytic (*A*), fatty acid (*B*), and Krebs cycle and oxidative phosphorylation (*C*), metabolic pathways. RNA was isolated from WT and SRC-2 KO hearts (n = 5). Individual gene expression is analyzed by ΔΔCt method with 18S RNA expression used as a normalizer and expression relative to WT. *D*, Lactate levels in WT and SRC-2 KO mouse heart tissue lysates. * = p≤0.05, ** = p≤0.01, and *** = p≤0.001. (Slc2a1- facilitated glucose transporter member 1 (GLUT1)^#^, Slc2a4- facilitated glucose transporter member 4 (GLUT4)^#^, Ldhd- lactate hydrogenase isoform d^#^, Pfkfb1- 6-phosphofructo-2-kinase/fructose-2,6-bisphosphatase 1^#^, Fbp2- fructose 1,6-bisphophatase 2^#^, Pkm2- pyruvate kinase, muscle, Pdk2 and 4- pyruvate dehydrogenase kinase 2^#^ and 4^#^, Fabp5, 7, and 1- fatty acid binding proteins 5^#^, 7^#^, and 1^#^, CD36- fatty acid transporter, AcsL1- acyl-CoA synthetase long-chain 1; Dgat 1 and 2- diacylglycerol O-acyltransferase 1 and 2, Acadl- long chain acyl-Coenzyme A dehydrogenase, Idh2- isocitrate dehydrogenase 2, Fh1- fumarate hydratase 1, ATPaf2- ATP synthase mitochondrial F1 complex assembly factor 2, COX4- cytochrome c oxidase subunit IV isoform 1, ATP5b- ATP synthase F1β subunit). A # indicates a gene identified in the microarray.

Coupled with the ability of SRC-2 to regulate glucose and fatty acid metabolism in other tissues [28], [29], [30], [31], and the identification of several metabolic pathways that are enriched from loss of SRC-2, we hypothesized that SRC-2 may be a regulator of cardiac metabolism. Several key glycolytic enzymes were up regulated from loss of SRC-2 in the microarray analysis. These results were confirmed through targeted gene expression analysis along with several other glycolytic regulators, suggesting that loss of SRC-2 leads to a metabolic phenotype mimicking that of a pressure overload-induced stressed heart (Figure 2A). In accordance with this phenotype, we also observed decreased expression of several of the major regulatory genes of fatty acid uptake, processing and storage, and breakdown (Figure 2B) and of several enzymes in both the Krebs cycle and oxidative phosphorylation pathways (Figure 2C). Increased lactate levels in the SRC-2 KO hearts support these gene expression changes (Figure 2D). Interestingly, immunoblot analyses show that SRC-2 expression peaks during the first week of life (Figure 1B), the period of development that coincides with the metabolic shift of the heart from the primary use of glucose to that of fatty acids for fuel [9].

### The Metabolic Gene Profile Switch is Accompanied by Sarcomeric and Stress Response Gene Expression Changes in the SRC-2 KO Heart

**Figure 3:**
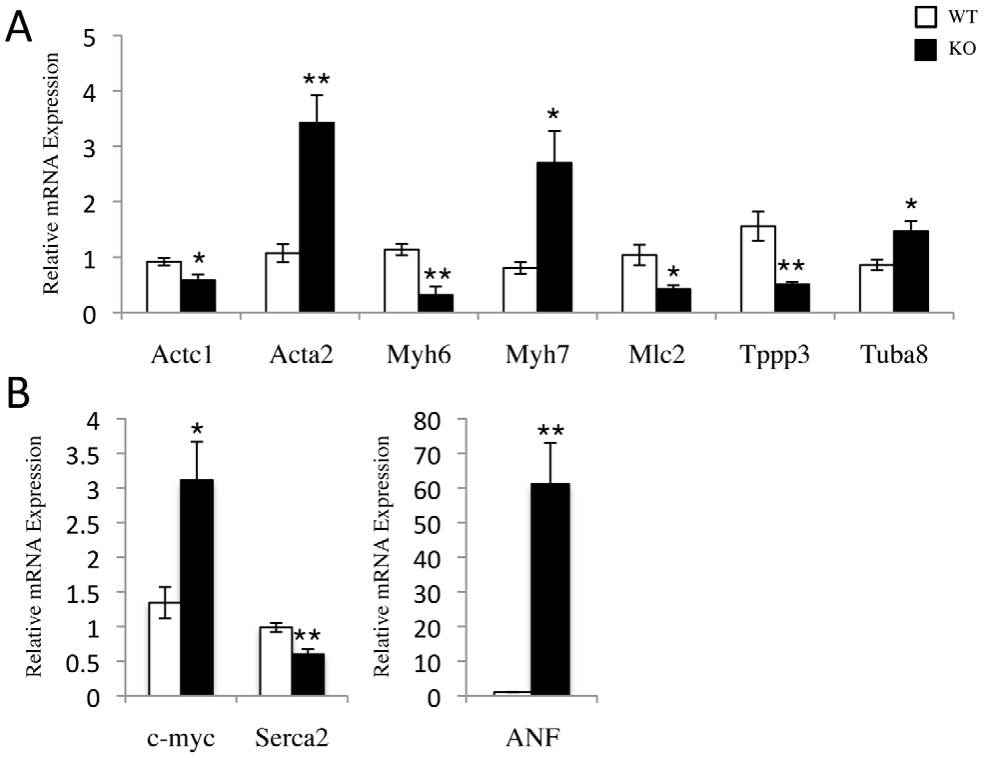
Shifts in sarcomeric and stress response gene expression profiles of SRC-2 KO. *A–B*, qPCR analysis of the indicated actin, myosin, and tubulin isoforms (*A*), and cardiac stress response (*B*) genes. RNA was isolated from WT and SRC-2 KO hearts (n = 5). Individual gene expression is analyzed by ΔΔCt method with 18S RNA expression used as a normalizer and expression relative to WT. * = p≤0.05 and ** = p≤0.01. (Actc1- cardiac actin, Acta2- smooth muscle actin^#^, Myh-myosin heavy chain 6 and 7, Mlc- myosin light chain (Myl2v), Tppp3- tubulin polymerization promoting protein family member 3^#^, Tuba8- tubulin, alpha 8^#^, c-myc- myelocytometosis oncogene, ANF- atrial natiuretic factor, Serca 2- cardiac muscle Ca+2 transporting ATPase). A # indicates a gene identified in the microarray.

Metabolic changes mimicking a stressed heart may also reflect changes in other cardiac pathways that respond transcriptionally to stress. We analyzed expression of several sarcomeric genes in the WT and SRC-2 KO hearts that were either identified in the microarray analysis or are commonly regulated during stress. Similar to that of a stressed heart, we observed major changes in the sarcomeric gene expression profile including major actin isoforms (*Actc1 and Acta2*), myosin heavy chain isoforms (decreased *Myh6,* increased *Myh7*), myosin light chain isoforms (*Mlc2*), tubulin isoforms (*Tuba8*), and tubulin-polymerization effector (*Tppp3*) alterations (Figure 3A). Furthermore, SRC-2 KO hearts have increased expression of *c-myc*, *ANF*, and decreased *Serca2*, which all are altered similarly during heart stress (Figure 3B).

### Expression of Several Key Transcription Factors is Altered SRC-2 KO Hearts

**Figure 4:**
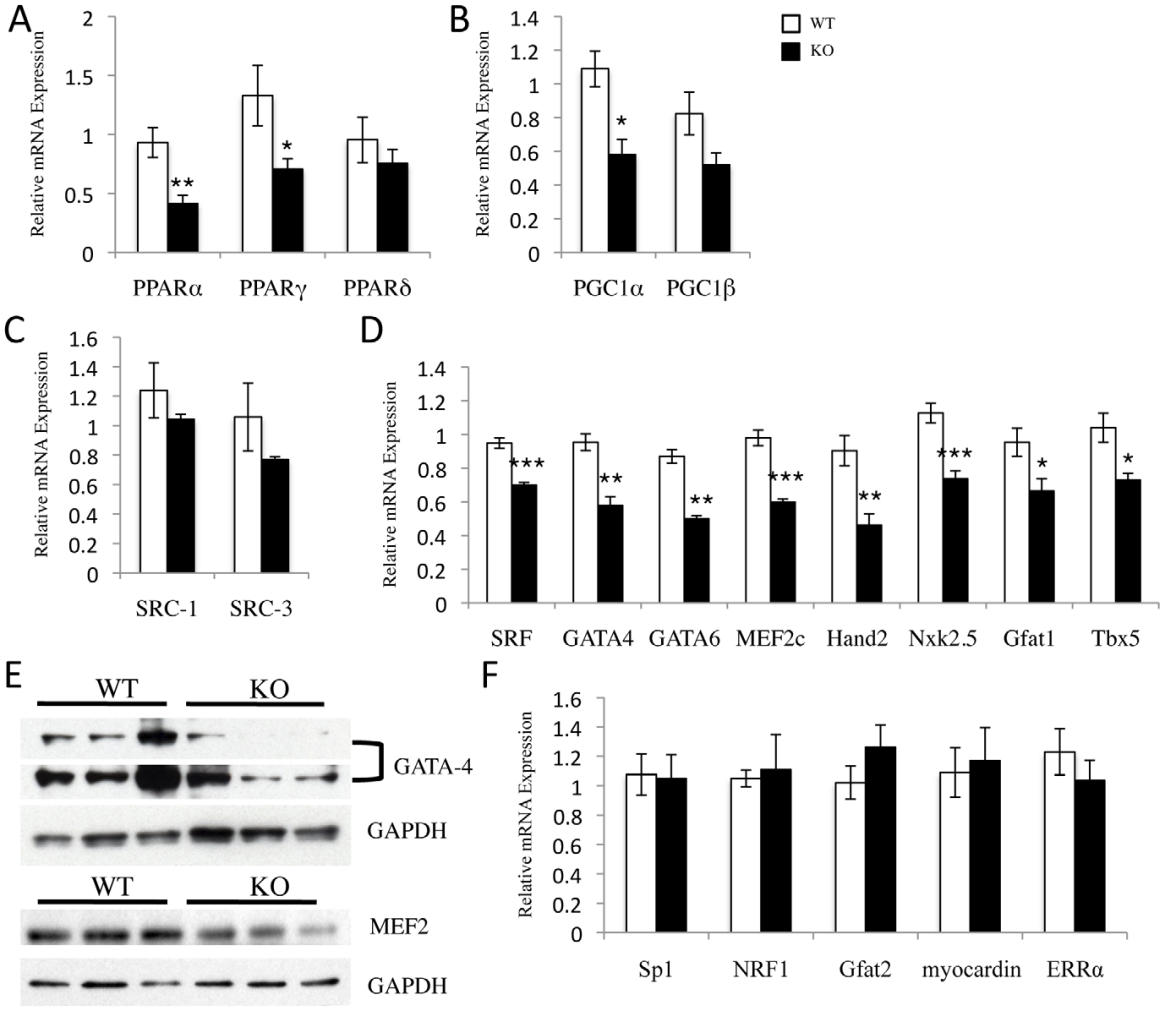
Loss of SRC-2 results in decreased expression of several cardiac transcription factors important for controlling metabolic and sarcomeric gene expression. *A–D and F*, qPCR analysis of the indicated transcription factor and transcription co-activator genes. RNA was isolated from WT and SRC-2 KO hearts (n = 5). Individual gene expression is analyzed by ΔΔCt method with 18S RNA expression used as a normalizer and expression relative to WT.*E*, Immunoblot for GATA-4 and MEF2 protein expression in WT and SRC-2 KO heart tissue lysates (n = 3).* = p≤0.05, ** = p≤0.01, and *** = p≤0.001. (PPAR- peroxisome proliferator activated-receptor α, β/δ, and γ, PGC-1- peroxisome proliferator activated-receptor γ coactivator-1 α and β, SRC- steroid receptor coactivator 1 and 3, SRF- serum response factor, GATA- GATA binding protein 4 and 6, MEF2c- myocyte enhancer factor 2c, Hand 2- heart and neural crest derivatives 2, Nkx2.5- NK2 transcription factor related 5, Gfat- glutamine frustose-6-phosphate transaminase 1 and 2, Tbx5- T-box 5^#^, NRF1- nuclear respiratory factor 1, ERR- estrogen-related receptor α). A # indicates a gene identified in the microarray.

All of the gene expression changes in the developing and/or stressed heart are accompanied by changes in transcription factor activity and/or expression. As a transcriptional coactivator, we hypothesized that SRC-2 may be acting in concert with or to regulate expression of one or many of these factors. Targeted gene expression analysis uncovered a widespread down regulation of many cardiac transcription factors and coregulators including metabolic modulators PPARα and γ and PGC-1α (Figure 4A–B) and structural and signaling modulators SRF, GATA4, GATA6, MEF2c, Hand2, Nkx2.5, Gfat1, and Tbx5 (Figure 4D). Protein expression analyses for at least two of these factors, GATA-4 and MEF2, resemble the gene expression decrease in SRC-2 KO hearts (Figure 4E). Importantly, not all transcription factors showed altered expression, as Sp1, NRF1, Gfat2, and myocardin, and ERRα revealed no changes in gene expression (Figure 4F).

Previously, some consequences of loss of SRC-2 have been suggested to possibly result from increased activity by another family member, SRC-1, in skeletal muscle [31]. In order to test this as a possible mechanism in the heart, we analyzed expression of SRC-1 and SRC-3 in SRC-2 KO hearts and observed no significant compensatory increase in their expression (Figure 4D).

### Unstressed SRC-2 KO Animals Show no Detectable Impairment of Cardiac Function

**Figure 5:**
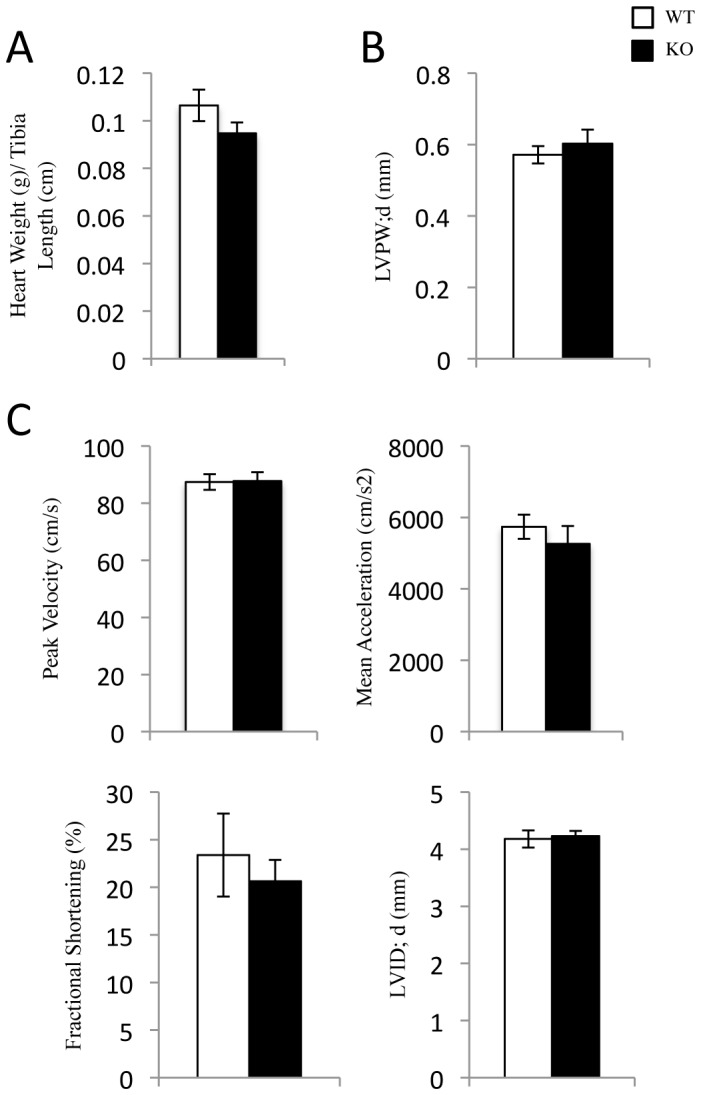
SRC-2 KO mice have no impairment in cardiac function in unstressed conditions. *A*, Total heart weight normalized to tibia length for WT and SRC-2 KO mice (n = 5). *B*, Diastolic left ventricle wall thickness measured by Echocardiography in WT and SRC-2 KO mice (WT n = 15, KO n = 12). *C*, Cardiac Doppler measurements of blood flow rates (peak velocity, mean acceleration, and fractional shortening) and diastolic left ventricle interior diameter measured by Echocardiography in WT and SRC-2 KO mice (WT n = 15, KO n = 12).

To assess whether the gene expression alterations resulting from SRC-2 loss affect cardiac function, we used echocardiography and cardiac Doppler analyses. We observed no significant change in physical parameters including total heart weight (Figure 5A), left ventricle diameter, or left ventricle wall thickness (Figure 5B and Table S3). Furthermore, there was no observed impairment of contraction based on mean acceleration, peak velocity, and fractional shortening in SRC-2 KO versus WT animals, nor were there any signs of left ventricular dilation in either group (Figure 5C and Table S3). Therefore, despite major changes in the gene expression profile, loss of SRC-2 does not largely affect steady-state cardiac function in the unstressed heart.

### Deletion of SRC-2 Increases the Decline in Cardiac Function in Response to TAC with an Absence of Hypertrophy

**Figure 6:**
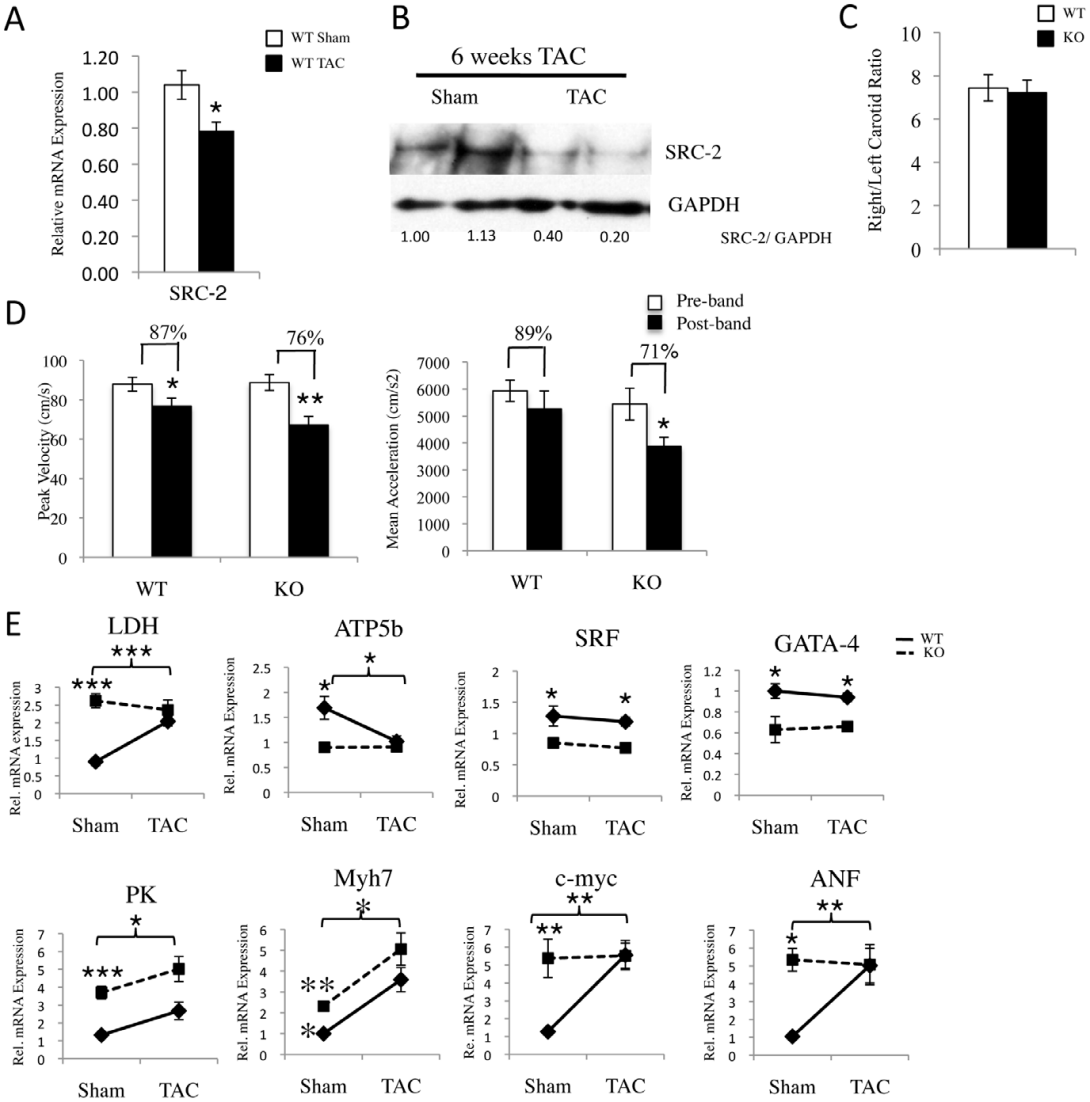
TAC in SRC-2 KO mice results in both greater decline of cardiac function and failure to hypertrophy. *A*, qPCR analysis of SRC-2 in WT sham and banded animals after 6 weeks of TAC (n = 4). Individual gene expression is shown normalized to 18S RNA expression. *B*, Immunoblot analysis of SRC-2 protein levels in sham and banded animals after 6 weeks TAC. GAPDH is used as a loading control. Numbers under the blot indicate densitometry measurements of SRC-2 versus GAPDH expression for each mouse. * = p≤0.05. *C,* Ratio of cardiac Doppler measurements of flow rates for right and left carotid to analyze amount of restriction due to TAC in WT and SRC-2 KO mouse groups (WT n = 16, KO n = 14). *D*, Cardiac Doppler measurements of blood flow rates (peak velocity and mean acceleration) in WT and SRC-2 KO hearts before and after 6 weeks TAC (WT n = 11, KO n = 7). Percent change as a result of banding is shown for each group. All Echo and Doppler “pre-” measurements are the same as presented in Figure 5. *E*, qPCR analysis of sham and TAC WT and SRC-2 KO hearts 6 weeks post-TAC for the indicated genes (n = 4). Individual gene expression is analyzed by ΔΔCt method with 18S RNA expression used as a normalizer and expression relative to WT. * = p≤0.05, ** = p≤0.01 and *** = p≤0.001.

Since ablation of SRC-2 results in a gene expression profile similar to that observed in a pressure overload-induced stressed heart, we hypothesized that SRC-2 KO hearts may either be primed for the stress and have improved function during TAC or, alternatively, these animals may not be able to further compensate for increased energetic demand from TAC, leading to impaired cardiac function. To test these ideas, we performed TAC in WT and SRC-2 KO animals and assessed their cardiac functions after 6 weeks. In accordance with human heart failure studies [1], we observed a moderate decrease in SRC-2 mRNA in TAC animals (Figure 6A), which corresponds with a decrease in SRC-2 protein expression 6 weeks post-TAC (Figure 6B). Due to the degree of variability in restriction, and thus imposed stress, that can occur from TAC, we found it important to compare pre-TAC (Figure 5) to post-TAC measurements in WT and SRC-2 KO animal groups. Importantly, both sets of animals were subjected to similar levels of aortic constriction (Figure 6C) and there was no decreased function observed in Sham control animals (Table S3). In the experimental groups, the SRC-2 KO animals showed decreased systolic cardiac function under TAC in relation to WT animals after 6 weeks of constriction. This decline in function is most evident through decreased peak velocity and mean acceleration as measured by cardiac Doppler (Figure 6D and Table S4). Diastolic function in the SRC-2 KO animals remained relatively intact, and at 6 weeks neither WT or SRC-2 KO animals observed major decreases in fractional shortening or increased left ventricular dilation suggesting these animals were not approaching heart failure at this time point (Table S4). At the molecular level, as expected, analysis of *LDH*, *ATP5b*, and *PK* demonstrated that the glycolytic program was up regulated in WT animals due to the TAC imposed stress. Furthermore, expression of these genes all increased to levels approaching those observed in the SRC-2 KO mice, which are relatively unchanged with TAC (Figure 6E). Similarly, *MHCβ (Myh7)* is increased in both WT and SRC-2 KO mice, with ending WT levels being similar to unstressed SRC-2 KO levels. Representative analysis of cardiac transcription factors *SRF* and *GATA4* indicate that these remain decreased independent of stress in the SRC-2 KO mice. As described by others [8], WT mice show increased expression of stress-induced genes *c-myc* and *ANF* with TAC, resulting in levels similar to those observed in control SRC-2 KO mice without TAC (Figure 6E).

### SRC-2 KO Animals Show Disruption of Several Hypertrophic Pathways

**Figure 7:**
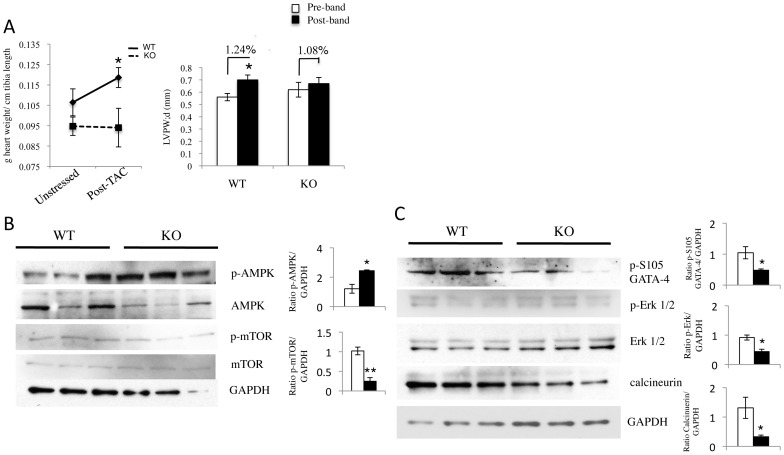
Hypertrophic signaling pathways are impaired in SRC-2 KO hearts. *A,* Echocardiography analysis of diastolic left ventricle wall thickness before and after 6 weeks TAC in WT and SRC-2 KO mice (WT n = 11, KO n = 7). Percent change as a result of banding is shown for each group. Measurement of WT and SRC-2 KO total heart weight normalized to tibia length in unstressed animals and post- 6 weeks TAC (WT n = 11, KO n = 7). All Echo and Doppler “pre-” measurements are the same as presented in Figure 5. *B-C*, Immunoblot analysis of the indicated proteins in WT and SRC-2 KO heart tissue lysates made on hearts 6 weeks post-TAC. GAPDH is used as a loading control. Each graph represents quantitation of the blot for each lane compared to its own GAPDH and then normalized to one WT sample (n = 3). * = p≤0.05 and ** = p≤0.01.

Somewhat surprisingly, while WT animals showed the normal hypertrophic response through increased left ventricular wall thickness and increased total heart weight (Figure 7A and Table S4), SRC-2 KO hearts had a blunted response. This decreased hypertrophic response in the SRC-2 KO animals led us to investigate activation of several pathways previously shown to be important for the cardiac growth observed during the hypertrophic response, including metabolic regulation of mTOR activity and transcriptional control of other components. Metabolic pathway analysis revealed increased phosphorylated AMPK and a corresponding decrease in phosphorylated mTOR in SRC-2 KO animals after TAC (Figure 7B) as would be expected during blunted hypertrophic signaling. On the transcriptional side, several other pathways appear to be down regulated in SRC-2 KO hearts including activation of GATA-4 via phosphorylation at S105 and activation of its upstream activator Erk1/2 (Figure 7C) as well as controller of an alternate hypertrophic pathway, calcineurin (Figure 7C).

## Discussion

The shift to a fetal gene program observed in the unstressed SRC-2 KO hearts suggests that SRC-2 activity is important in controlling adult metabolic and sarcomeric gene expression profiles. These pathways are major targets of remodeling during pressure overload-induced cardiac stress, highlighting SRC-2 as a novel component of the stress response. Despite already mimicking the gene expression profile of a stressed heart, SRC-2 KO mice have decreased systolic cardiac performance in response to TAC. We propose that this decreased function results from a lack of coordination of the major stress responsive pathways so that integration of these pathways to maintain function is impaired. A large contributor is likely increased metabolic stress from the onset of pressure overload since metabolic changes are the first sensors of stress [8]. Importantly, the data suggest that induction of the fetal/stressed gene program in the adult mouse does not necessarily accompany decreased cardiac function, but can predispose the heart to cardiac decline with increased stress, despite being able to avoid the lag time between stress onset and transcriptional remodeling. Furthermore, the lack of hypertrophy observed in the SRC-2 KO mice independent of expression of fetal myosin and actin isoforms prior to TAC, implies a dissociation between sarcomeric isoform expression and hypertrophic response and indicates that these pathways are at least somewhat separately regulated and suggests that mis-coordination of these pathways may lead to decreased cardiac function.

### SRC-2 KO Mice Exhibit Activation of the Fetal Gene Program

We observe gene expression remodeling due to loss of SRC-2 in both the metabolic and sarcomeric pathways implying that SRC-2 is an important upstream regulator of both of these key cardiac pathways. While expression of the metabolic fetal gene profile in the adult mouse heart has been previously shown in response to loss of a metabolic enzyme itself, such as Acsl1 [42], or loss of a upstream regulator of metabolic enzyme expression such as loss of the PPAR family of transcription factors [43], complete remodeling of both the metabolic and sarcomeric gene expression programs has not been reported under unstressed conditions. Furthermore, we observed decreased expression of several cardiac transcription factors in the SRC-2 KO hearts, many of which have been previously characterized to regulate metabolism or sarcomeric gene expression, but usually not both. For example, loss of PPARα or PPARγ is sufficient to drive cardiac metabolic disruption and impairment of fatty acid use [11], [13], but there is little known about their effects on sarcomeric gene expression. Conversely, GATA-4 has been shown to regulate sarcomeric and stress-response genes such as α-actin and atrial naturietic factor [44], but has not been shown to directly regulate extensive metabolic gene expression. Further, along with other described roles for SRC-2 in regulating transcription of important metabolic genes in other tissues [28], [29], [30], [31], this report highlights SRC-2 as a possible regulator of coordinated metabolic regulation on a whole body level. Since these studies were performed with a germline KO of SRC-2, it is important to remember that not all genes altered are direct targets of SRC-2, but that these are changes acquired over time due to changes in the direct SRC-2 targets. Future studies investigating these direct targets will provide critical information for the mechanism of the cardiac transcription program. Together, our data describes SRC-2 as a novel controller of these pathways and suggests that this control is through modulation of expression of many known regulators of these pathways, the transcription factors themselves.

Remodeling of adult cardiac gene expression to a fetal expression profile is associated with pressure overload, ischemia, and aging. This switch in metabolic and sarcomeric gene expression is postulated to help compensate for the energetic demands of the increased stress, although it is unclear whether maintaining the switch under long-term conditions is beneficial or detrimental to cardiac function [9]. Interestingly, despite the remodeling resulting from loss of SRC-2, there is no decrease in heart function under unstressed conditions, suggesting that the switch to the fetal gene program is at least sufficient to meet steady-state energy demands. However, upon additional stress, in the current study via pressure overload from TAC, mice lacking SRC-2 had a more rapid decline in heart function suggesting that the fetal gene expression program is not sufficient to meet the change in energetic demand of a heart under increased workload due to TAC.

### SRC-2 Ablation Prevents Compensatory Hypertrophic Response

One striking result is the lack of hypertrophy in response to TAC in SRC-2 KO mice. We have found that several pathways involved in the hypertrophic response are disrupted in TAC SRC-2 KO hearts (Figure 7), including altered metabolic signaling and an impaired transcriptional response. First, upon TAC, SRC-2 KO mice have increased activation of metabolic ATP sensor, AMP kinase (AMPK), as well as active mTOR, a downstream target of AMPK (Figure 7). AMPK regulation of mTOR inhibits protein synthesis, a main contributor to hypertrophy [20]. We suggest that this loss of regulation results from an inability of the SRC-2 KO mice to meet the energetic demands of cardiac hemodynamic overload since they are already relying on an altered metabolic pathway (Fig. 2) and that this results in an ATP deficiency upon TAC, leading to inhibition of hypertrophy and decreased cardiac function. Second, the hypertrophic response also has a strong transcriptional component, in which several transcription factors have been shown to participate. We observe decreased transcriptional signaling through upstream modulators Erk1/2 and calcineurin and direct regulation of transcription factor activity through phosphorylation of GATA-4 at S105. Decreased activity of all of these pathways has previously been shown to down regulate and/or prevent the hypertrophic response [24], [25]. Due to the multi-faceted nature of the hypertrophic response we cannot differentiate the relative contributions of these pathways.

### Loss of SRC-2 Disrupts the Integrated Pathways of the Stress Response

Our observation that stress-responsive actin and myosin isoform switching can be uncoupled from hypertrophy in the SRC-2 KO mice is supported by recent observations suggesting that *Myh7* expression can be uncoupled from hypertrophic stimuli at the cellular expression level (reviewed in [10]) and that adult isoforms can be down regulated without corresponding increases in fetal isoforms during hypertrophy in the rat heart [45]. The idea that these changes in sarcomeric gene expression, so frequently observed together with hypertrophy, can be dissociated from one another suggests an important element of timing involved with the stress response. We speculate that when this timing is disrupted there can be elimination of the adaptive response or decreased efficiency gained from the response, ultimately resulting in decreased cardiac function, as is observed in the SRC-2 KO animals. These data suggest a new layer of complexity to be considered in control of the cardiac stress response.

In conclusion, we describe SRC-2 as a novel regulator of cardiac function by showing that loss of SRC-2 in the mouse results in extensive remodeling of the adult cardiac transcriptome to one that resembles the fetal or stressed heart. With left ventricular pressure overload *in vivo*, the absence of SRC-2 results in decreased cardiac function with a surprising suppression of hypertrophy. Taken together with previous observations of SRC-2 expression changes during cardiac failure in humans [1], our results strongly suggest roles for SRC-2 in regulating the metabolic, sarcomeric and hypertrophic pathways suggest that regulation of SRC-2 is an important novel component in the three main adaptive programs during cardiac stress. Furthermore, our data raise the possibility that integration of these multiple pathways in a temporal sequence is critical in the adaptive stress response.

## Supporting Information

Table S1 **Genes significantly altered in SRC-2 KO heart microarray.** Microarray analysis was performed on WT and SRC-2 KO heart tissue (WT, KO n = 3). Data and statistical analyses were performed as described in the *Methods.* Significance for input genes was defined by FDR<0.05. Genes are ordered according to heat map clustering in *Fig. 1A*.
(DOCX)

Table S2 **GO Pathway Enrichment for genes changed in SRC-2 KO Heart Microarray.** Pathway enrichment analysis was performed on genes identified with altered expression from a microarray comparison of WT and SRC-2 KO heart tissue. Significance for input genes was defined by FDR<0.15. Pathways are ordered by p-value. Analysis was performed using the Gene Set Enrichment Analysis software (GSEA).
(DOCX)

Table S3 **Cardiac Measurements on Pre- and Post-TAC Sham animals.** Cardiac Doppler and Echocardiography measurements taken on Sham WT (n = 4) and SRC-2 KO (n = 5) mice before and after TAC (6–10 weeks). LVI- Left Ventricular Interior diameter, FS- Fractional Shortening, LA- Left Atrium, BW- Body Weight.
(DOCX)

Table S4 **Cardiac Measurement on Pre- and Post-TAC experimental animals at 6 weeks.** Cardiac Doppler and Echocardiography measurements taken on Sham WT (n = 11) and SRC-2 KO (n = 7) mice before and after TAC (6 weeks). LVID- Left Ventricular Interior diameter, FS- Fractional Shortening, LVPW- Left Ventricular Posterior Wall thickness, LA- Left Atrium, BW- Body Weight.
(DOCX)
