# Supplementary figures and images for: SRC-2 Coactivator Deficiency Decreases Functional Reserve in Response to Pressure Overload of Mouse Heart
Source: PLoS One. 2012 Dec 31;7(12):e53395. doi: 10.1371/journal.pone.0053395 (PMC3534027; doi:10.1371/journal.pone.0053395)

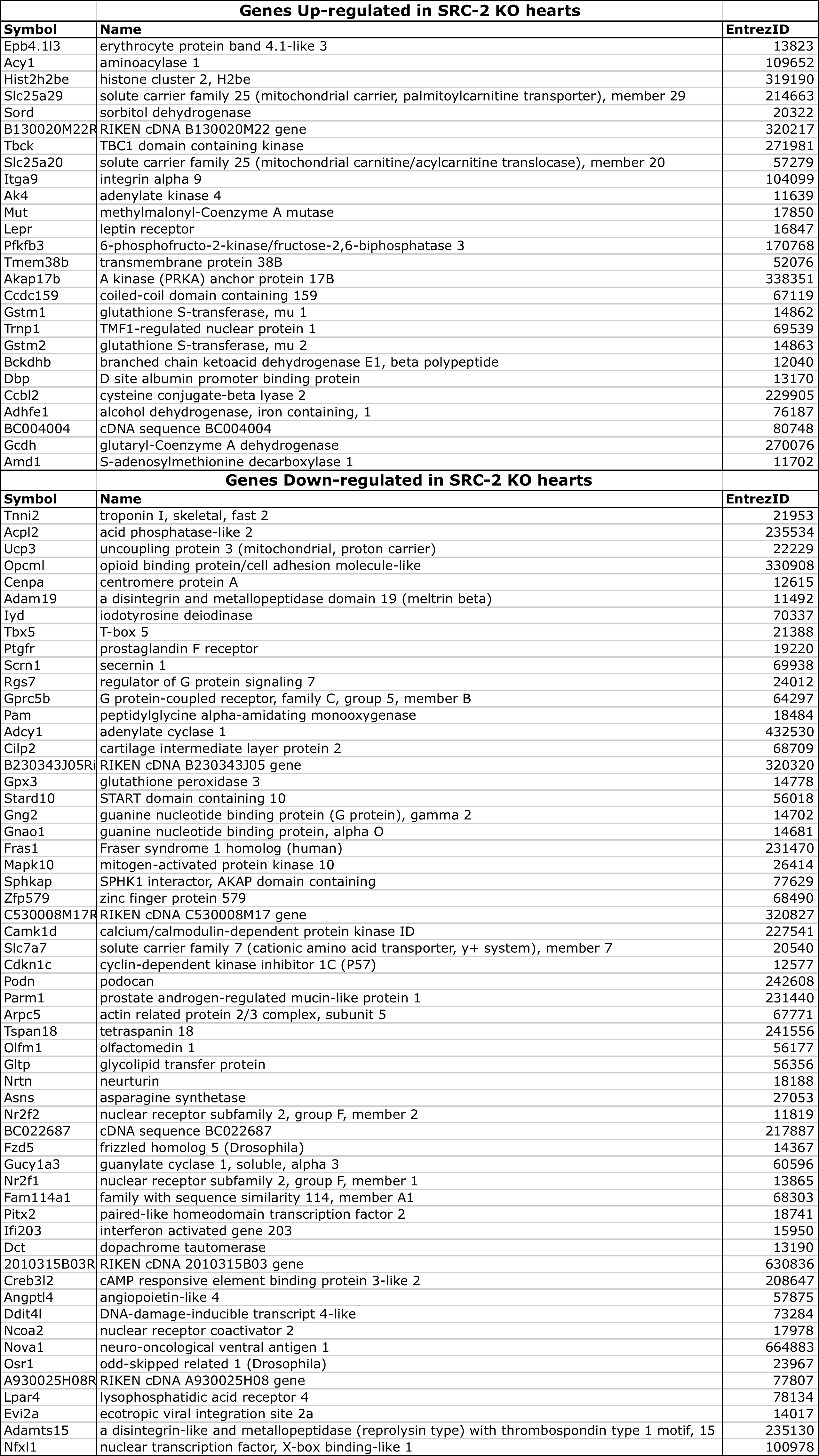
Table S1. Genes significantly altered in SRC-2 KO heart microarray.

Supplement: Table S1 — Genes significantly altered in SRC-2 KO heart microarray. Microarray analysis was performed on WT and SRC-2 KO heart tissue (WT, KO n = 3). Data and statistical analyses were performed as described in the Methods. Significance for input genes was defined by FDR<0.05. Genes are ordered according to heat map clustering in Fig. 1A . (DOCX) [file pone.0053395.s001.docx]

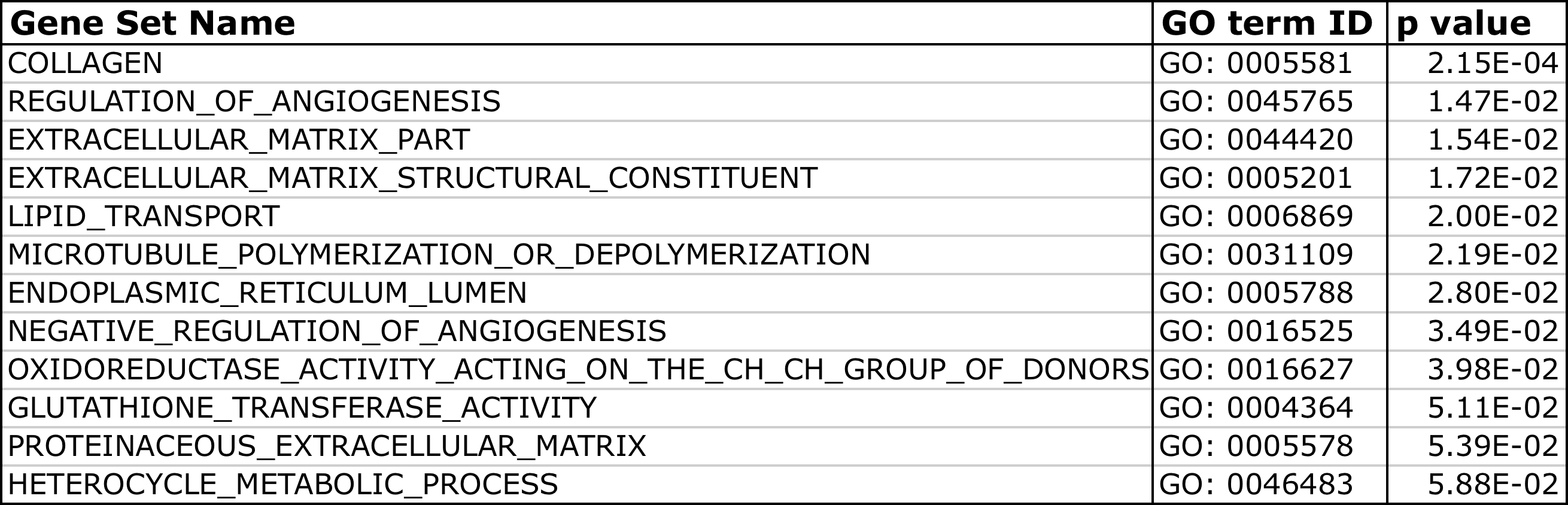
Table S2. GO Pathway Enrichment for genes changed in SRC-2 KO Heart Microarray.

Supplement: Table S2 — GO Pathway Enrichment for genes changed in SRC-2 KO Heart Microarray. Pathway enrichment analysis was performed on genes identified with altered expression from a microarray comparison of WT and SRC-2 KO heart tissue. Significance for input genes was defined by FDR<0.15. Pathways are ordered by p-value. Analysis was performed using the Gene Set Enrichment Analysis software (GSEA). (DOCX) [file pone.0053395.s002.docx]

Supplemental Table 3. Cardiac Measurements on Pre- and Post-TAC Sham animals.


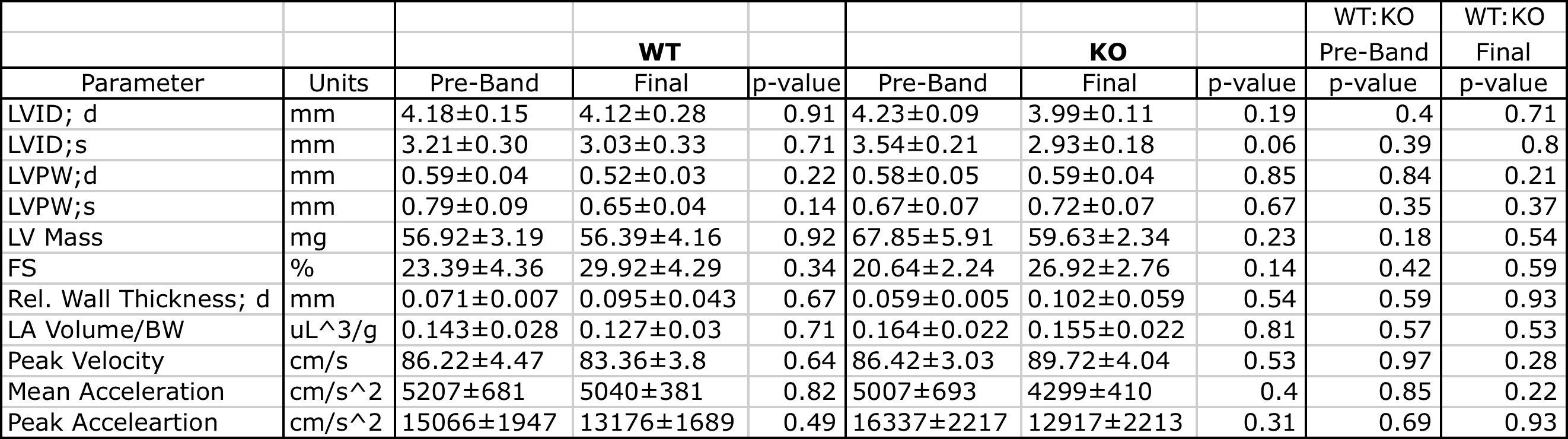

Supplement: Table S3 — Cardiac Measurements on Pre- and Post-TAC Sham animals. Cardiac Doppler and Echocardiography measurements taken on Sham WT (n = 4) and SRC-2 KO (n = 5) mice before and after TAC (6–10 weeks). LVI- Left Ventricular Interior diameter, FS- Fractional Shortening, LA- Left Atrium, BW- Body Weight. (DOCX) [file pone.0053395.s003.docx]

Table S4. Cardiac Measurement on Pre- and Post-TAC experimental animals at 6 weeks


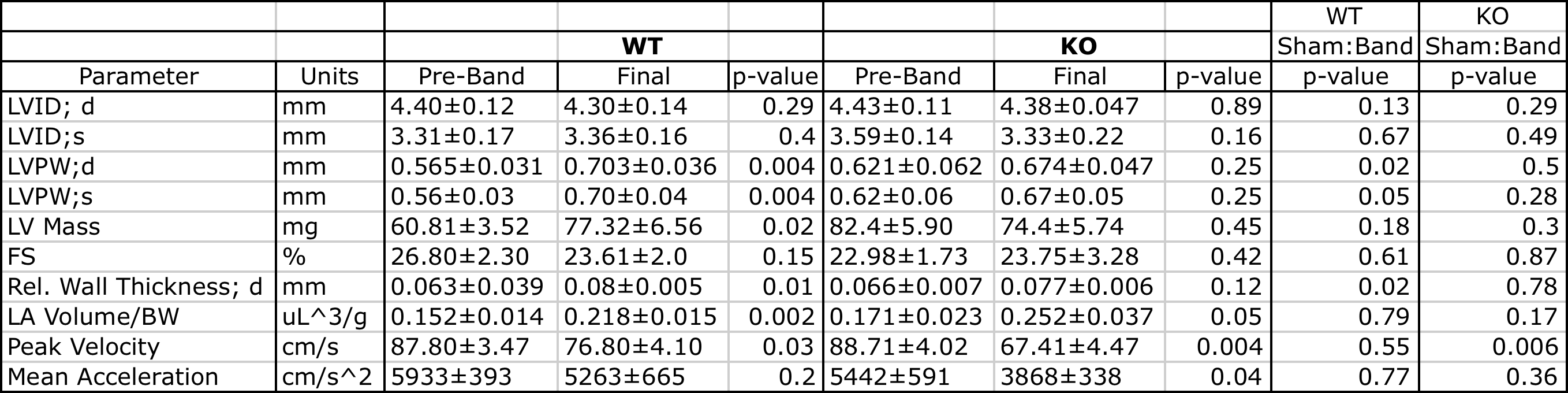

Supplement: Table S4 — Cardiac Measurement on Pre- and Post-TAC experimental animals at 6 weeks. Cardiac Doppler and Echocardiography measurements taken on Sham WT (n = 11) and SRC-2 KO (n = 7) mice before and after TAC (6 weeks). LVID- Left Ventricular Interior diameter, FS- Fractional Shortening, LVPW- Left Ventricular Posterior Wall thickness, LA- Left Atrium, BW- Body Weight. (DOCX) [file pone.0053395.s004.docx]
